# Empiric guideline-recommended weight-based vancomycin dosing and mortality in methicillin-resistant *Staphylococcus aureus bacteremia: a retrospective cohort study*

**DOI:** 10.1186/1471-2334-12-104

**Published:** 2012-04-27

**Authors:** Ronald G Hall, Christopher A Giuliano, Krystal K Haase, Kathleen A Hazlewood, Chistopher R Frei, Nicolas A Forcade, Sara D Brouse, Todd Bell, Roger J Bedimo, Carlos A Alvarez

**Affiliations:** 1 Department of Pharmacy Practice, Texas Tech University Health Sciences Center, School of Pharmacy, Dallas, USA; 2 Department of Clinical Sciences, University of Texas Southwestern, Dallas, USA; 3 Department of Pharmacy Practice, Texas Tech University Health Sciences Center, School of Pharmacy, Amarillo, USA; 4 Division of Pharmacotherapy, University of Texas, Austin, USA; 5 Department of Internal Medicine, Texas Tech University Health Sciences Center, School of Medicine, Amarillo, USA; 6 University of Texas Southwestern, Department of Internal Medicine, Dallas, USA; 7 University of Wyoming School of Pharmacy, Swedish Family Medicine Residency Program, Englewood, USA; 8 University of Kentucky Healthcare, Lexington, USA; 9 Wayne State University Eugene Applebaum College of Pharmacy and Health Sciences, Detroit, USA; 10 Mission Regional Medical Center, Mission, USA

**Keywords:** Weight, Obesity, Efficacy, MRSA, Vancomycin, Bacteremia

## Abstract

**Background:**

No studies have evaluated the effect of guideline-recommended weight-based dosing on in-hospital mortality of patients with methicillin-resistant *Staphylococcus aureus* bacteremia.

**Methods:**

This was a multicenter, retrospective, cohort study of patients with methicillin-resistant *Staphylococcus aureus* bacteremia receiving at least 48 hours of empiric vancomycin therapy between 01/07/2002 and 30/06/2008. We compared in-hospital mortality for patients treated empirically with weight-based, guideline-recommended vancomycin doses (at least 15 mg/kg/dose) to those treated with less than 15 mg/kg/dose. We used a general linear mixed multivariable model analysis with variables identified a priori through a conceptual framework based on the literature.

**Results:**

A total of 337 patients who were admitted to the three hospitals were included in the cohort. One-third of patients received vancomycin empirically at the guideline-recommended dose. Guideline-recommended dosing was not associated with in-hospital mortality in the univariable (16% vs. 13%, OR 1.26 [95%CI 0.67-2.39]) or multivariable (OR 0.71, 95%CI 0.33-1.55) analysis. Independent predictors of in-hospital mortality were ICU admission, Pitt bacteremia score of 4 or greater, age 53 years or greater, and nephrotoxicity.

**Conclusions:**

Empiric use of weight-based, guideline-recommended empiric vancomycin dosing was not associated with reduced mortality in this multicenter study.

## Background

Vancomycin is commonly used for the treatment of methicillin-resistant *Staphylococcus aureus* (MRSA) bacteremia. The United States of America Food and Drug Administration (FDA) originally approved a dosing regimen of 1 gram administered intravenously (IV) every 12 hours (or 500 mg IV every six hours). The FDA approved dosing regimen has not changed in over 50 years despite several studies demonstrating that vancomycin pharmacokinetics in adults are best predicted by actual body weight [1-4]. Dosing handbooks that are used in clinical practice continue to recommend a fixed dose for all patients regardless of weight [5,6].

Over the past decade, there has been an increase in the incidence of MRSA strains with vancomycin minimum inhibitory concentration (MIC) values ≥ 1 μg/ml as well as heteroresistant vancomycin-intermediate *S. aureus*. In response to increasing vancomycin MICs in MRSA isolates, the Infectious Diseases Society of America, the American of Society of Health-Systems Pharmacists, and the Society of Infectious Diseases Pharmacists developed weight-based dosing recommendations for vancomycin based on pharmacokinetic and pharmacodynamic data (15–20 mg/kg/dose IV administered every eight to 12 hours) [7]. The clinical effectiveness of this empiric weight-based, guideline-recommended dosing regimen (at least 15 mg/kg/dose) has yet to be critically evaluated.

Data evaluating guideline-recommended weight-based vancomycin dosing are needed to confirm the efficacy of this approach versus lower traditional dosing. Therefore, we conducted a multicenter retrospective cohort study to evaluate the effectiveness of guideline-recommended weight-based dosing for vancomycin on mortality of patients with MRSA bacteremia.

## Methods

### Study location and patients

We identified a retrospective cohort of patients admitted with MRSA bacteremia (using microbiological records) between 01/07/2002 and 30/06/2008 at three types of hospitals (400 bed urban, 200 bed veteran affairs, and 604 bed university). Patients were included if they were at least 18 years old and had received parenteral vancomycin for at least 48 hours. Patients were excluded if, at the time of the first vancomycin dose, they were receiving dialysis, had a creatinine clearance of 30 ml/min or less based upon the Cockcroft-Gault equation, received prior vancomycin within the hospital stay, had a culture-proven MRSA infection in the previous six months, or were pregnant [8]. The institutional review board of each respective site (North Texas Veterans Health Care System, Texas Tech University Health Sciences Center, and University of Texas Health Science Center, San Antonio) approved the study and waived the need for informed consent.

### Study design and data collection

We conducted a retrospective cohort study of patients who received guideline-recommended weight-based dosing with those receiving lower dosing of vancomycin for MRSA bacteremia. Our primary outcome was in-hospital mortality.

### Study definitions

All definitions were selected prospectively during the initial trial design. Guideline-recommended weight-based dosing was defined as at least 30 mg/kg/day in the first 24 hours (at least 15 mg/kg/day for patients with a creatinine clearance of 30–50 ml/min). Lower dosing was defined as receiving less than 30 mg/kg/day (less than 15 mg/kg/day for patients with a creatinine clearance of 30–50 ml/min). Pitt bacteremia score was calculated based on the date when the first positive blood culture was obtained [9,10]. In-hospital mortality was defined as patient death occurring within the index hospital stay. Nephrotoxicity was defined as an increase in creatinine by more than 0.5 mg/dl or greater than a 50% increase from baseline on two consecutive days [7].

### Statistical analysis

Recursive partitioning was used to ascertain significant cut-points in continuous candidate variables associated with an increased risk of mortality [11]. Univariable associations were explored using either Chi-square or Fisher’s Exact tests. A Pitt bacteremia score cutoff of 4 or higher was used based on previous literature demonstrating significantly higher sensitivity and specificity for predicting severity of illness [12]. A vancomycin trough 15 mcg/ml or greater was based on the guideline recommended trough concentration range of 15–20 mcg/ml [7]. Variables examined in the initial univariable analysis included receipt of guideline recommended weight-based vancomycin dosing, intensive care unit (ICU) admission, age 53 years, Pitt bacteremia score of 4 or higer, vancomycin trough 15 mcg/ml or greater, nephrotoxicity, Charlson comorbidity index score of 5 or higher, weight of 100 kg or greater, and gender.

Variables identified as significant through univariable analysis (*p* c 0.1) and those conceptually regarded as biologically reasonable causes of mortality were considered for inclusion in the multivariable model. A generalized linear mixed-effect model was utilized to identify independent predictors of mortality. Hospital site was treated as a random effect whereas other covariates were treated as fixed effects. Variables were retained in the multivariable model if their respective p values were < 0.05. Adjusted ORs and 95% confidence intervals were calculated for each variable. Effect measure modification and biologic interaction were also extensively evaluated. All analyses were performed using SAS 9.2 (Cary, North Carolina) and RTREE (Available at: http://c2s2.yale.edu/software.rtree).

## Results

### Patients

A total of 798 patients with MRSA bacteremia were evaluated, with 337 included in the study cohort after application of the exclusion criteria. In-hospital mortality data were not collected for one patient. The baseline characteristics of the cohort are shown in Table 1. Forty-seven patients (14%) died during their hospital stay. Survivors were more likely to be male and less likely to have vancomycin started in the ICU (34 vs. 83%). Survivors also had a younger median age, higher baseline renal function, lower Charlson Comorbidity Index, and a less severe Pitt Bacteremia score. Vancomycin was empirically dosed according to guidelines in 33% of survivors and 38% of non-survivors (*p* = 0.48) with a median initial daily dose of 24.9 vs. 21.0 mg/kg/day (*p* = 0.036), respectively. Vancomycin was adjusted for renal function in 39% of survivors and 59% of non-survivors (*p* = 0.028).

**Table 1:** **Baseline characteristics of the cohort**^**A**^

**Characteristic**	**Survivors (n = 289)**	**Non-survivors (n = 47)**	***p*-value**
Male gender (%)	77%	94%	0.009
Age (years)	53 (42, 63)	65 (56, 77)	<0.001
Race (%)			0.66
Caucasian	64%	74%	
African American	15%	11%	
Hispanic	18%	13%	
Other	3%	2%	
Height (cm)	175.0 (165.1, 180.3)	175.0 (169.5, 183.0)	0.14
Weight (kg)	78.1 (65, 95.6)	80.0 (63.0, 99.0)	0.91
Serum Creatinine (mg/dl)	0.90 (0.7, 1.2)	1.10 (0.7, 1.5)	0.035
Creatinine Clearence (ml/min)	86.9 (61.0, 122)	64.0 (42.9, 86.3)	<0.001
Pitt Bacteremia Score	1 (0, 3)	3 (2, 6)	<0.001
Charlson Comorbidity Index	2 (1, 3)	2 (2, 4)	0.030
Guideline-recommended Vancomycin Dose Received (%)	33%	38%	0.48
Dose adjusted for renal function (%)	39%	59%	0.028
Vancomycin Started in Intensive Care Unit (%)	34%	83%	<0.001
Initial Vancomycin Dose (mg/kg/day)	24.9 (19.6, 30.4)	21.0 (17.0, 29.0)	0.036
Nephrotoxins (%)			
Intravenous Contrast	34%	13%	0.004
Aminoglycosides	19%	19%	0.98
Vasopressors	7%	36%	<0.001
Infection source (%)			<0.001
Bloodstream catheter-related	18%	36%	
Central nervous system	0.4%	0%	
Gastrointestinal	0.7%	0%	
Osteomyelitis	0.7%	4%	
Pulmonary	15%	36%	
Skin/muscle	36%	6%	
Other	20%	17%	

A = Results are presented as median (interquartile range) unless otherwise noted.

The primary sources of bacteremia were skin/soft tissue (32.5%), intravenous catheter (20.2%), and pulmonary (17.8%). Other documented sources included genitourinary (8%), bone (1.2%), gastrointestinal (0.5%), and central nervous system (0.3%). The source of bacteremia was not specified for 19.8% of patients. Non-survivors were less likely to have a skin/muscle source of their bacteremia and more likely to have a bloodstream catheter-related or pulmonary source. Seventy-nine patients (23.4%) received concomitant antimicrobials active against MRSA. Non-survivors were less likely to receive IV contrast dye and more likely to receive vasopressors. Nineteen percent of both groups received aminoglycosides. Nephrotoxicity occurred in 82 patients (23.4%).

The overall median (interquartile range) length of hospital stay was 16 (9, 32) days for survivors and 23 (14, 56) for non-survivors (*p* = 0.009). The median length of stay after vancomycin initiation was 14 (7.5, 26.5) days for survivors and 19 (9, 42) for non-survivors. Thirty-two percent of patients had a hospital stay longer than 28 days. The median ICU length of stay was 0 (0, 4) days for survivors and 9 (3, 39) for non-survivors.

### Univariable and multivariable analysis

In the univariable analysis (Table 2), mortality was similar among patients who received guideline-recommended dosing versus lower dosing (16% vs. 13%, OR 1.26 [95%CI 0.67-2.39]). Factors that increased risk for mortality by univariable analysis included: male gender, age of 53 years or greater, ICU admission, Pitt bacteremia score of four or greater, nephrotoxicity, and vancomycin trough of 15 mcg/ml or greater.

**Table 2:** Univariable analysis of risk factors for mortality

**Variable**	**Odds Ratio**	**95% Confidence Interval**
Guideline-Recommended Vancomycin Dosing	1.26	0.67-2.39
Intensive Care Unit Admission	9.50	4.27-21.12
Age of 53 years or greater	7.89	3.04-20.52
Pitt Bacteremia Score of four or greater	4.47	2.34-8.54
Vancomycin trough of 15 mcg/ml or greater	2.48	1.30-4.74
Nephrotoxicity	4.95	2.60-9.42
Charlson Comorbidity Index of five or greater	1.63	0.75-3.54
Weight of 100 kg or greater	1.14	0.55-2.37
Male gender	4.43	1.33-14.71

Vancomycin dosing also was not significantly associated with mortality (OR 1.05, 95%CI 0.48-2.27) when retained in the multivariable analysis (Table 3). The only factors that remained significant in the multivariable model were age of 53 years or greater, ICU admission, Pitt bacteremia score of four or greater, and nephrotoxicity.

**Table 3:** Multivariable analysis of independent risk factors for mortality

**Variable**	**Odds Ratio**	**95% Confidence interval**
Guideline-Recommended Vancomycin Dosing	0.71	0.33-1.55
Intensive Care Unit Admission	6.14	2.46-15.35
Age of 53 years or greater	5.58	1.88-16.51
Pitt Bacteremia Score of four or greater	2.93	1.21-7.09
Nephrotoxicity	2.29	1.05-4.97
Vancomycin trough of 15 mcg/ml or greater	1.24	0.56-2.75

## Discussion

To our knowledge, this is the first published multicenter study to evaluate the effect of empiric guideline-recommended weight-based dosing on mortality in MRSA bacteremia. We did not observe any significant relationship between empiric guideline-recommended weight-based vancomycin dosing and in-hospital mortality. Independent predictors of in-hospital mortality were ICU admission, Pitt bacteremia score of 4 or greater, age 53 years or greater, and nephrotoxicity.

Most studies evaluating the effectiveness of vancomycin dosing for MRSA infections have compared high vs. low vancomycin trough concentrations. Our results parallel the findings of these studies. A recently published prospective cohort study assessing patients with MRSA pneumonia and bacteremia failed to demonstrate that achieving vancomycin trough concentrations to 4 to 5 times the MIC were associated with any difference in clinical response rates [13]. Another study of 102 patients evaluated the association between vancomycin trough concentrations and mortality in MRSA pneumonia; 11% of patients had concomitant bacteremia [14]. This study did not find a difference in vancomycin trough concentrations between survivors and non-survivors (13.6 vs. 13.9 mcg/ml). Using vancomycin trough concentrations to evaluate mortality is potentially confounded by the fact that vancomycin is eliminated by the kidneys. Acute renal failure is an independent risk factor for mortality, which also results in increased vancomycin trough concentrations (regardless of the cause of acute renal failure).

Our findings suggest that other patient factors play are more important factors in determining in-hospital mortality for patients with MRSA bacteremia than vancomycin dosing. The empiric use of guideline-recommended weight-based dosing in the absence of compelling efficacy or effectiveness data has the potential to place patients at an increased risk of concentration-related adverse events with an uncertain benefit to the patient. Clinicians should critically evaluate vancomycin trough concentrations and kidney function to help minimize this risk in patients receiving guideline-recommended weight-based vancomycin regimens.

The implications of this study on clinical practice are limited by its retrospective design, use of in-hospital mortality, lack of MIC data, and lack of data regarding time to a therapeutic vancomycin trough concentration. Utilizing in-hospital mortality as our primary endpoint resulted in fewer events than utilizing 30 day mortality or vancomycin failure [15-17]. This difference created greater variability in the multivariable model and limited the number of candidate variables evaluated in the multivariable model. In spite of this limitations, the factors associated with mortality in this study are similar to others [15]. The study’s retrospective design allowed a more realistic estimate of clinical effectiveness than could be observed in a randomized, controlled trial. Increased vancomycin MIC values may have also played a role in the lack of benefit associated with guideline-recommended dosing. However, clinicians rarely have vancomycin MIC data available when selecting an empiric vancomycin dose. We did not collect data regarding the time to the first therapeutic vancomycin trough, as quicker times to therapeutic trough concentrations may improve vancomycin efficacy. However, this information may have been biased in this retrospective evaluation by clinician judgment regarding when trough concentrations were ordered. Our results may have also been subject to a selection bias that patients weighing greater than 70 kilograms were less likely to receive guideline-recommended, weight-based vancomycin dosing since most patients (85%) received 1 gram every 12 hours. Furthermore, the implications of loading doses on mortality need further evaluation since none of the study institutions utilized this dosing strategy.

## Conclusions

In our study, we did not observe any significant association between guideline-recommended dosing and mortality. These results, in concert with the findings of others, call for a prospective, randomized comparison of empiric use of guideline-recommended weight-based vs. traditional dosing of vancomycin to ensure that patients treated with vancomycin receive doses that maximize efficacy and safety.

## Competing interests

Grant funding from AstraZeneca, Ortho-McNeil Janssen, and Pfizer: CRF, Scientific Advisory Board for Tibotec Therapeutics and Gilead Sciences: RJB, None: RGH, CAA, CAG, KKH, KAH, NAF, SDB, TB

## Authors’ contributions

RGH, CAG, KKH, KAH, SDB, RJB were involved in the study concept and design. RGH, CAG, CAA, CRF were involved in the data analysis and interpretation. RGH, CAG, KKH, KAH, CAA, CRF, NAF, SDB, TB, RJB were involved in the drafting of the manuscript for important intellectual content and had final approval of the manuscript.

## Pre-publication history

The pre-publication history for this paper can be accessed here:

http://www.biomedcentral.com/1471-2334/12/104/prepub
